# Molecular and pharmacological aspects of piperine as a potential molecule for disease prevention and management: evidence from clinical trials

**DOI:** 10.1186/s43088-022-00196-1

**Published:** 2022-01-28

**Authors:** Amit Kumar Tripathi, Anup Kumar Ray, Sunil Kumar Mishra

**Affiliations:** 1 Molecular Biology Unit, Institute of Medical Science, Banaras Hindu University, Varanasi, 221005 India; 2 Clinical Research Division, School of Basic and Applied Science, Galgotias University, Gautam Buddha Nagar, UP India; 3 Department of Pharmaceutical Engineering and Technology, Indian Institute of Technology (Banaras Hindu University, Varanasi, 221005 India; 4 Department of Pharmacognosy, I.T.S College of Pharmacy, Ghaziabad, UP 201206 India

**Keywords:** Anticancer, Piperine, Pharmacokinetics, Extractions, Clinical trials, COVID-19, Gut microbiota

## Abstract

**Background:**

Piperine is a type of amide alkaloid that exhibits pleiotropic properties like antioxidant, anticancer, anti-inflammatory, antihypertensive, hepatoprotective, neuroprotective and enhancing bioavailability and fertility-related activities. Piperine has the ability to alter gastrointestinal disorders, drug-metabolizing enzymes, and bioavailability of several drugs. The present review explores the available clinical and preclinical data, nanoformulations, extraction process, structure–activity relationships, molecular docking, bioavailability enhancement of phytochemicals and drugs, and brain penetration properties of piperine in the prevention, management, and treatment of various diseases and disorders.

**Main body:**

Piperine provides therapeutic benefits in patients suffering from diabetes, obesity, arthritis, oral cancer, breast cancer, multiple myeloma, metabolic syndrome, hypertension, Parkinson's disease, Alzheimer’s disease, cerebral stroke, cardiovascular diseases, kidney diseases, inflammatory diseases, and rhinopharyngitis. The molecular basis for the pleiotropic activities of piperine is based on its ability to regulate multiple signaling molecules such as cell cycle proteins, anti-apoptotic proteins, P-glycoprotein, cytochrome P450 3A4, multidrug resistance protein 1, breast cancer resistance protein, transient receptor potential vanilloid 1 proinflammatory cytokine, nuclear factor-κB, c-Fos, cAMP response element-binding protein, activation transcription factor-2, peroxisome proliferator-activated receptor-gamma, Human G-quadruplex DNA, Cyclooxygenase-2, Nitric oxide synthases-2, MicroRNA, and coronaviruses. Piperine also regulates multiple signaling pathways such as Akt/mTOR/MMP-9, 5′-AMP-activated protein kinase-activated NLR family pyrin domain containing-3 inflammasome, voltage-gated K+ current, PKCα/ERK1/2, NF-κB/AP-1/MMP-9, Wnt/β-catenin, JNK/P38 MAPK, and gut microbiota.

**Short conclusion:**

Based on the current evidence, piperine can be the potential molecule for treatment of disease, and its significance of this molecule in the clinic is discussed.

**Graphical abstract:**

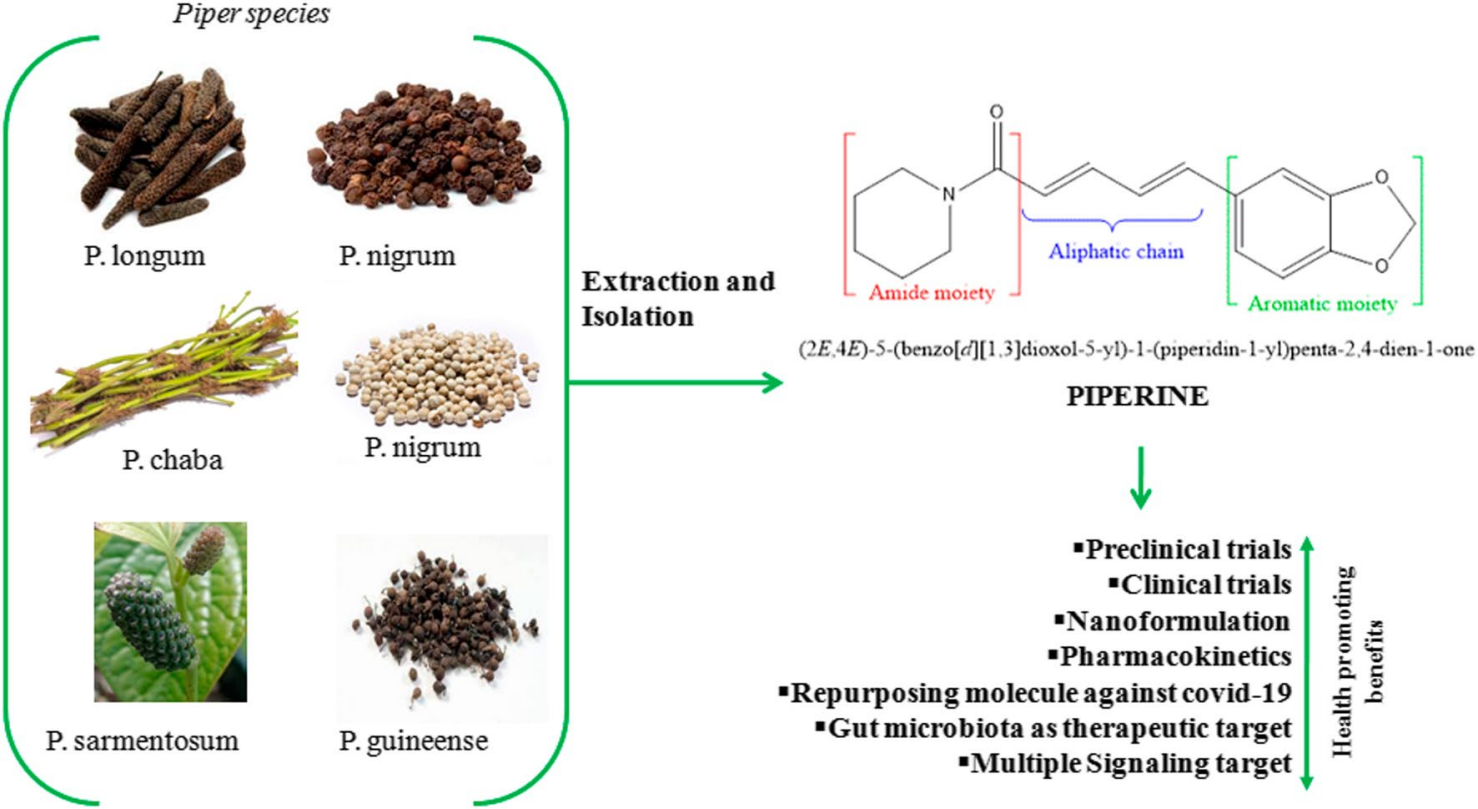

## Background

Piperine (1-[5-[1,3-benzodioxol-5-yl]-1-oxo-2,4-pentadienyl]piperidine) is a nitrogen-containing alkaloid molecule, first isolated in the form of yellow crystalline solid (MW 285.33 g.mol^−1^, mp = 128–130 °C) by Danish chemist Hans Christian Orstedt in 1820 from the dried fruit extract of *pepper* [1]*.* Chemically, piperine molecules consist of conjugated aliphatic chains, which act as a connecting structure between piperidine and 5-(3, 4-methylenedioxyphenyl) moiety. Piperine occurs naturally in black, green, and white *pepper* (Table 1) [2–4]. Other alkaloids are also present in black pepper extracts such as piperanine, piperettine, piperylin A, piperolein B, and pipericine [5]. During the last two decades, piperine has received considerable attention for its beneficial health effects [6–8].

**Table 1:** Members of *Piperaceae* family containing piperine [9]

Name of plant	Part of plant	Piperine content (%)
*Piper nigrum*	Fruit	1.7–7.4
*Piper longum*	Spike and root	5–9
	Fruit	0.03
*Piper chaba*	Fruit	0.95–1.32
*Piper guineense*	Fruit	0.23–1.1
*Piper sarmentosum*	Root	0.20
	Stem	1.59
	Leaf	0.104
	Fruit	2.75

**Fig. 1:**
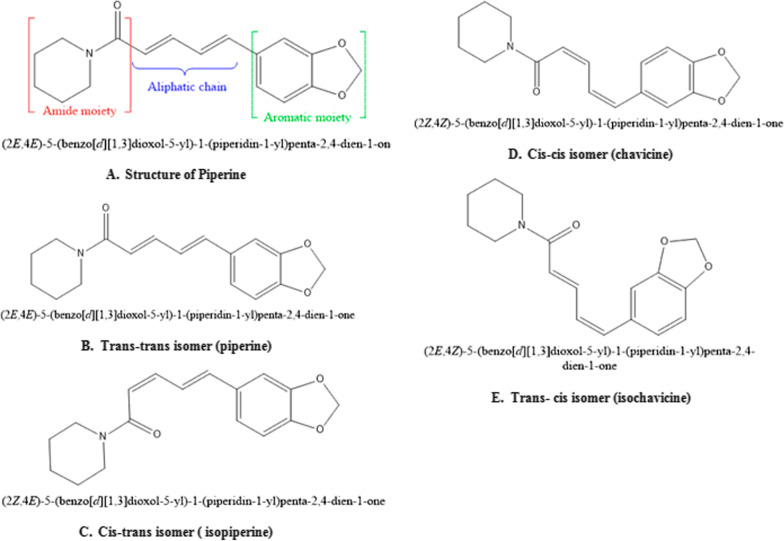
**A** Structure**; B–E** Isomers of piperine [5]

**Fig. 2:**
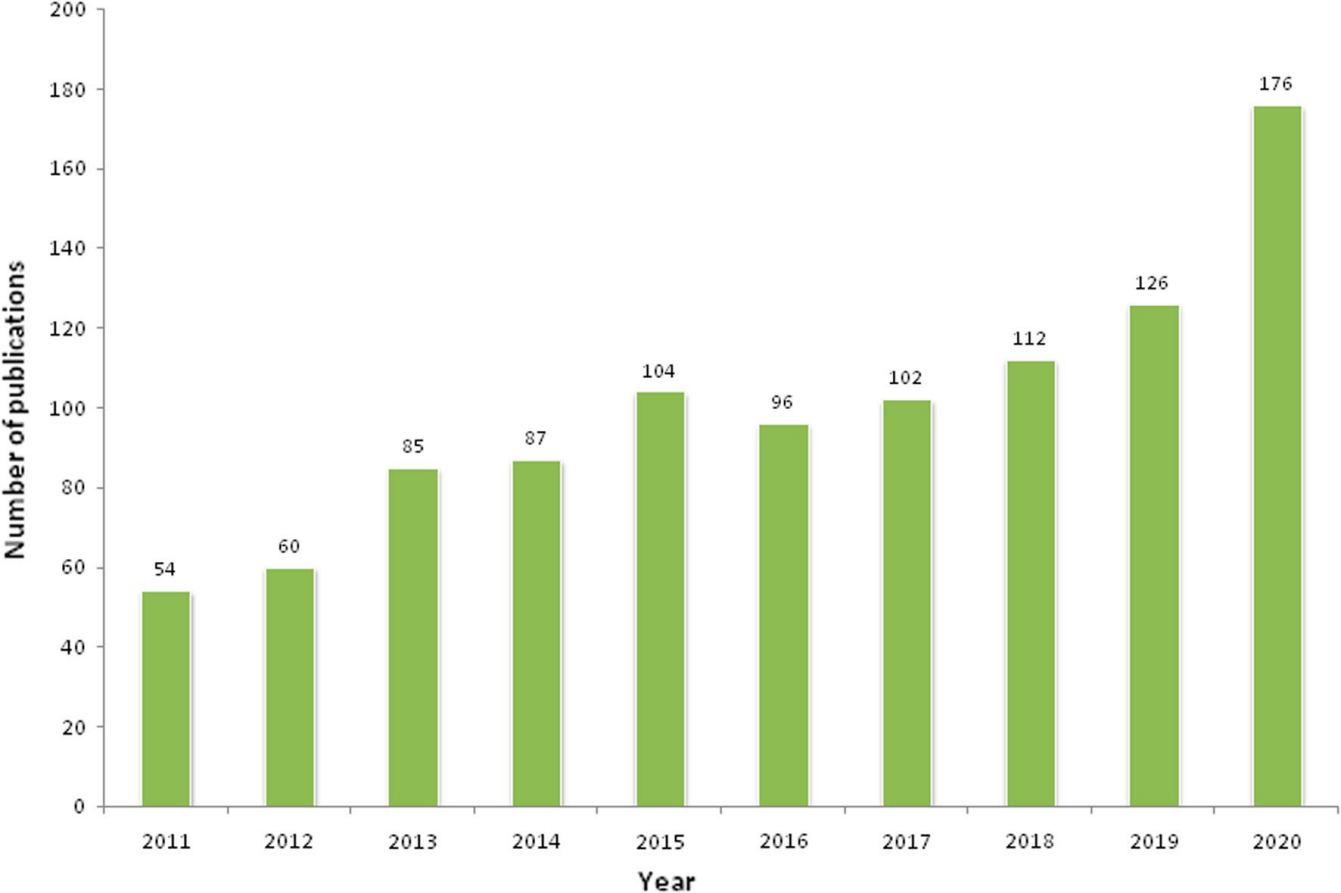
Total number of publications of piperine in previous ten years. Source: https://pubmed.ncbi.nlm.nih.gov/?term=PIPERINE&filter=datesearch.y_10&timeline=expanded

Naturally, piperine exists in four isomeric forms (Fig. 1) [9, 10]. However, only piperine isomers have pungency and biological activity compared to the other three. Other studies showed *cis* and *trans*-isomer of piperine possess significant anti-hepatotoxic as well as antioxidant effects [1]. Light-induced isomerization of piperine increases with light intensity and its exposure time [11]. Chemical synthesis of piperine was done by Ladenburg and Scholtz in 1894, by reaction of the piperic acid chloride with piperidine. The multiple biological activities of piperine have been demonstrated in both preclinical and clinical studies. The clinical trials completed are 11 and in addition that are currently ongoing are 5; a total of 1002 articles have been published on piperine in the last 10 years (Table 2 and Fig. 2) [12–15]. However, some of the clinical trial data is published but not registered.

**Table 2:** Clinical trials on piperine*

Condition & (number of patients)	Phases I, II, III or IV & (Status)	Dose, duration	Principal Investigator affiliation	Duration (months)	References
KOA (60)	I (Completed)	7.5 mg/day, 4 wks	Motahar Heidari-Beni, IUMS	Jan 2018–May 2018 (5)	Heidari-Beni et al. [154]
TCS 12)	I (Completed)	20 mg/day,10 days	S. K. Bedada**,** UCPSKU	2016–2016 –	Bedada et al. [155, 156]
MS (12)	I (Completed)	20 mg/day, 2 days	HMO & A. Hoffman and A. Domb, DRBCPHU	Aug 2013–Jan 2015 (17)	Cherniakov et al. [157]
AIDS (08)	I (Completed)	20 mg/day,7 days	Ravisekhar Kasibhatta, BCRPL, Hyderabad	2007–2008 –	Kasibhatta and Naidu [158]
NAFLD (79)	III (Completed)	5 mg/day, 8 weeks	Dr. Abasalt Borji, NUMS	Jan 2017–Nov 2017 (10)	Mirhafez et al. [159]
T2DM (100)	III (Ongoing)	5 mg/day, 12weeks		Jun 2015–Present (12)	*
NAFLD (70)	II (Ongoing)	5 mg/day, 12weeks		Jan 2018–Present –	
HIVS (60)	I (Completed)	–	Philip C Smith, School of Pharmacy, UNC Chapel Hill	Sep 2003–Mar 2006 (30)	
MN, Pain, BS, Urinary Urgency (09)	I (Active, not recruiting)	–	Aminah Jatoi, Mayo Clinic, Rochester, Minnesota, United States	Mar 2016–Mar 2021 (60)	
CKD (30)	NA (Recruiting)	500 mg of curcumin and piperine, 3 capsules/day, 12 weeks	Denise Mafra, Federal University Fluminense, Rio de Janeiro, Brazil	Oct 2020–Oct 2021 (12)	
Hair Thinning (70)	NA Recruiting)	95% piperine extract in formulation, 4 capsules/ day, 180 days	Glynis Ablon, ABSIRC, Manhattan Beach, California, United States	Jun 2019–Jan 2021 (18)	
Epilepsy (10)	I (Completed)	20 mg/day, 2 days	Smita Pattanaik, NOD-PGIMER, Chandigarh, India	2017–2017 –	Pattanaik et al. [160]
OD (40)	I&II (Completed)	Group1 = 150 μM Group 2 = 1 mM	Laia Rofes, GPLRU, Hospital de Mataro´, Spain	Jun 2011–Feb 2012 (9)	Rofes et al. [161]
MS (12)	I (Completed)	20 mg/day, 10 days	S K Bedada, UCPSKU	2016–2016 –	Bedada and Boga [162]
OA (53)	III (Completed)	15 mg/day, 6 weeks	Dr. Yunes Panahi, CIRC-BUC, CRDU-BH, BUMS, Iran	Jan 2011–Jan 2012 (12)	Panahi et al. [163–165]
Vitiligo (63)	II&III (Completed)	1% Topical solution, 12weeks	Anoosh Shafiee, SRC-SBUM, Iran	Jun 2016–Sep 2016 (3)	Shafiee et al. [15]

^*^ Referenced from:—US National Library of Medicine: https://www.clinicaltrials.gov/ct2/results?cond=&term=piperine&cntry=&state=&city=&dist = ; University hospital Medical Information Network-Clinical Trials Registry (UMIN-CTR): https://www.umin.ac.jp/ctr/index.htm & Iranian Registry of Clinical Trials: https://www.irct.ir/

Exploring the broad-spectrum bioactivities of piperine has been demonstrated over a decade that can be harnessed in agriculture as pesticide and medicinal use. The insecticidal properties of piperine have been first observed in 1924 [16]. The LD_50_ for piperine is 330 and 200 mg/kg for single intra-gastric and subcutaneous injections, respectively [16]. Piperine is also reported to inhibit enzymes (cytochrome P450, UDP-glucoronyltransferase) that catalyze the biotransformation of nutrients and drugs, thereby enhancing their bioavailability and *in vivo* efficacies [11]*.*

**Fig. 3:**
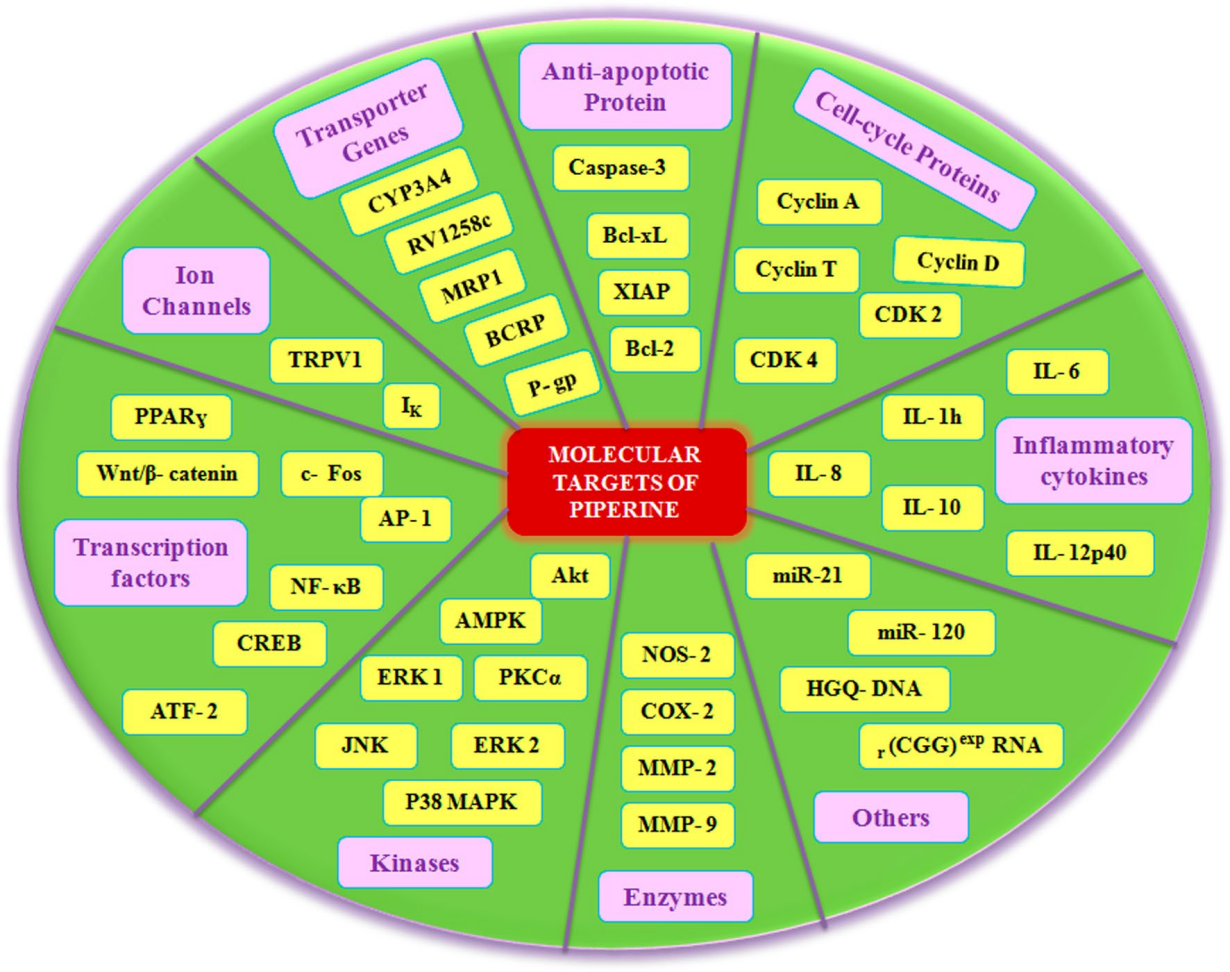
Targets of Piperine

**Fig. 4:**
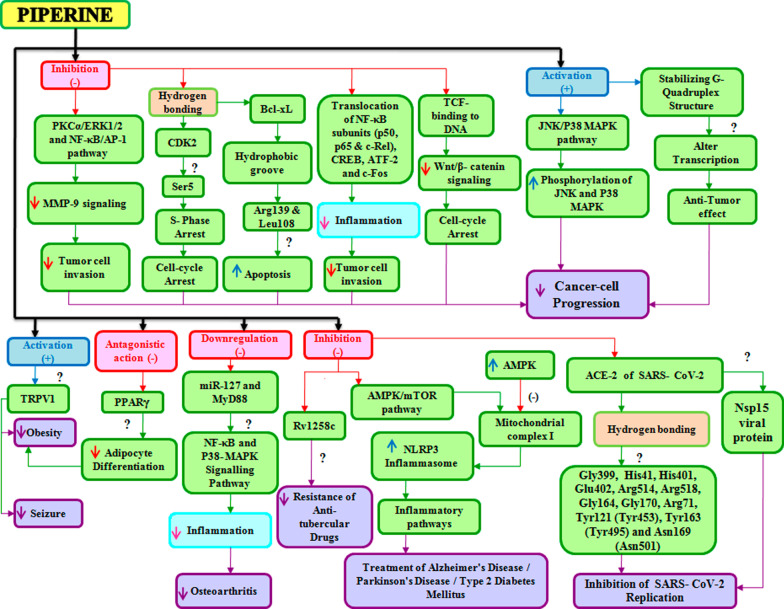
Proposed scheme for signaling molecular target of piperine

Clinical trials have looked into the protective and therapeutic effect of piperine against many diseases and disorders including hypertension, diabetes, cancer, neurological, cardiovascular, and reproductive as well as against microbial infections such as viral, bacterial, and fungal infections. Both clinical and preclinical data have shown that piperine has many targets (Figs. 3 and 4) and that it can modulate the various signaling molecules such as Wnt, NF-κB, cAMP response element-binding protein, activation transcription factor-2, peroxisome proliferator-activated receptor-gamma, human G-quadruplex DNA, cyclooxygenase-2, nitric 5.

oxide synthases-2, MicroRNA, SARS CoV-2, Akt/mTOR/MMP-9, AMPK-activated NLRP3 inflammasome, IK, ERK1/2, nuclear factor erythroid 2 like 2 (Nrf2) and r (CGG) exp RNA. The pleiotropic mechanistic action of piperine is therefore attributed to its ability to interact with a broad spectrum of molecular targets that include kinases, transcription factors, cell cycle proteins, inflammatory cytokines, receptors, and signaling molecules.

## Main text

### Chemical modification, structure–activity relationships, and synthetic analogs of piperine

Chemically, piperine is an alkaloid and the structure is composed of three subunits: An amide function constituted by a piperidine ring with α-β-unsaturated carbonyl moiety, a 1,3-benzodioxole group, also called piperonal nucleus and a butadiene chain (Fig. 1A). All four isomers of piperine showed inhibitory activity against *Leishmania donovani* pteridine reductase 1 **(**LdPTR1), while the maximum inhibitory effect was demonstrated by isochavicine. It was reported that piperine, isopiperine, isochavicine, activated both TRPV1 and TRPA1. Many studies have reported different types of derivatives and analogues of piperine **(**Table 3) along with their structure–activity relationship (SAR) and biological activities. The efficiency of piperine derivatives increases by replacing the piperidine moiety with *N,N*-dipropyl, *N,N*-diisopropyl, *N,N*-dibutyl, *p*-methyl piperidine, or *N,N* bis(trifluoromethyl) groups. Potency enhancers exchange the piperidine moiety with *N,N*-dibutyl, *N,N*-diisobutyl, or *N,N*-bis trifluoromethyl groups [17]. The most active piper amides are the N-isobutyl-substituted ones that resemble pesticidal activity. For the activity of piper amides, the lipophilic chain must contain at least four carbons and a conjugated bond adjacent to amide carbonyl with a bulky amine is necessary for binding, which makes piperine a model compound for the bioactive amides. Activity among the piperidine amides increases with increasing substitution on the piperidine ring carbons, with ethyl substituted being more active than the methyl analogues. Saturation of the side chain in piperine resulted in enhanced inhibition of Cytochrome P450 (CYP450), while modifications in the phenyl and basic moieties in the analogues produced maximal selectivity in inhibiting either constitutive or inducible CYP450 [18]. Several piperine derivatives with modifications at the piperidine moiety and the aliphatic chain have been reported to inhibit survivin protein, a small target in the inhibitor of apoptosis (IAP) family and regulator of cell division in cancer [19]. Few modified analogues of piperine showed promising activity on the TRPV1 and GABAA receptors [17, 20–24].

**Table 3:**
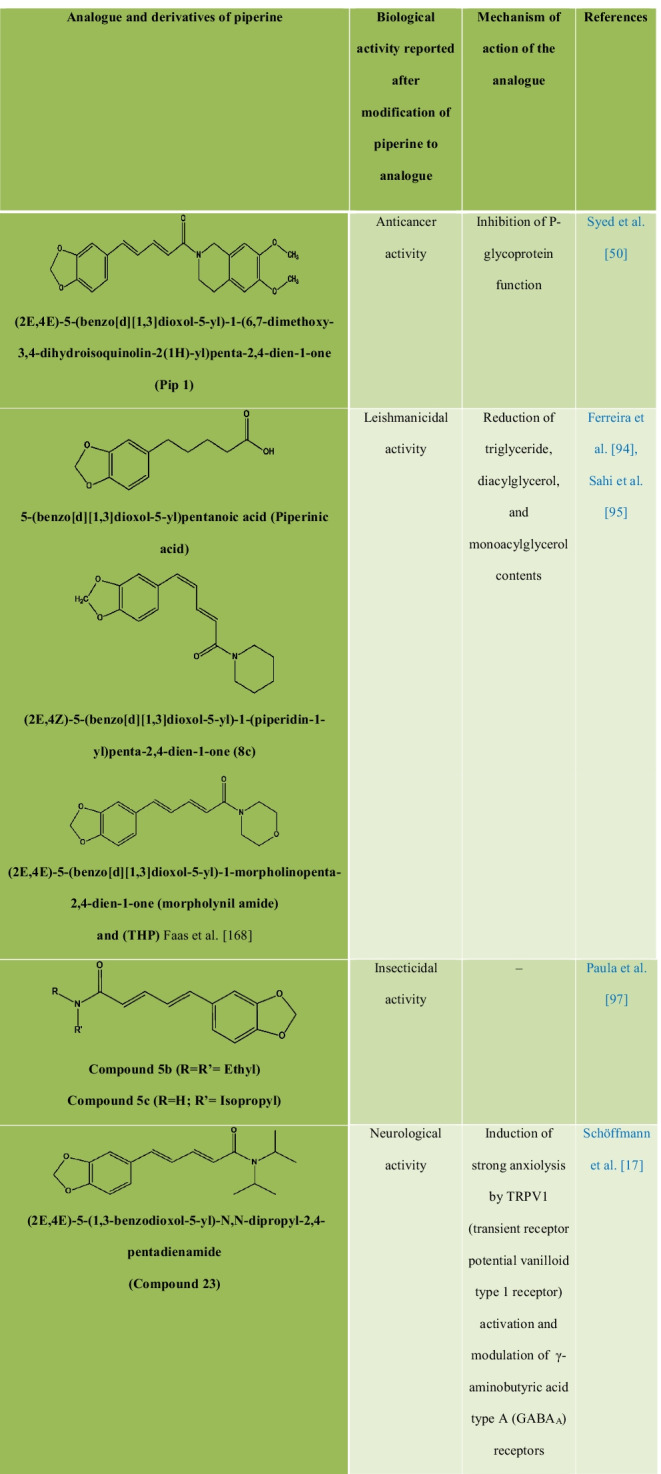

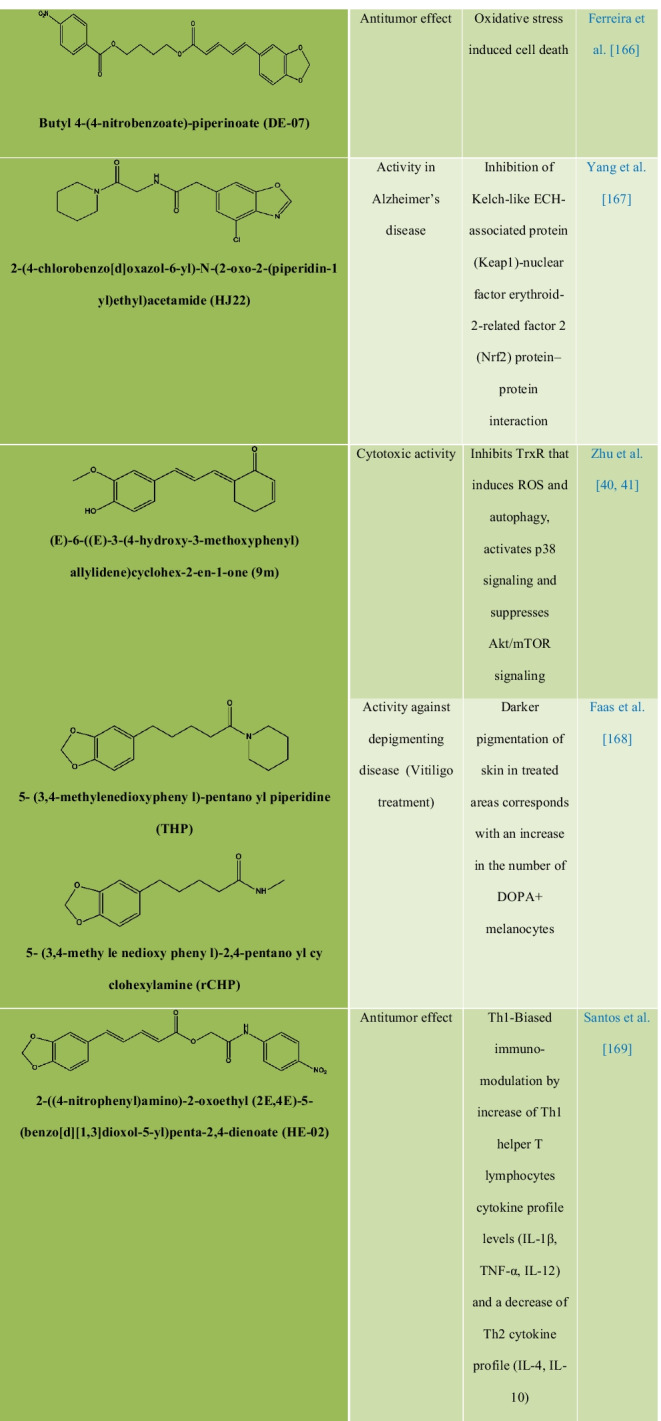

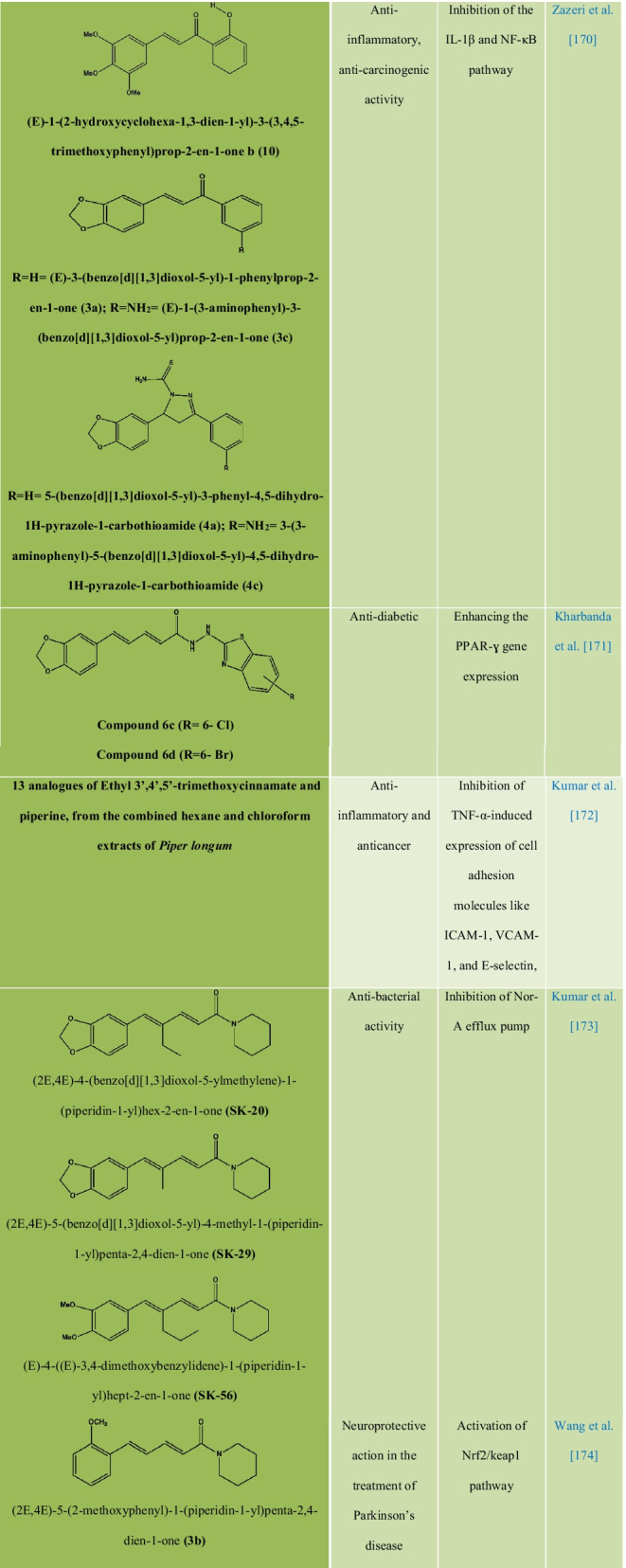
Analogues and derivatives of piperine with their biological activity and mechanism of action

### Extraction methods of piperine from black and white pepper

Piperine content varies in plants from the *Piperaceae* family from 2 to 7.4% in both black and white pepper [1]. Different methods are used to extract piperine (Table 4); these methods often suffer demerits such as inadequate extraction efficiency, photodegradation, tedious and expensive isolation methodology*.* It is therefore needed in the first place to determine the best factors and conditions to optimize these shortcomings [25]*.* Increasing the surface area of the pepper improved the efficiency of extraction by 109.02% [26]. The nonpolar solvents like petroleum ether brought the highest extraction efficiency of 94% with a purity of 85% [1]. The double bypasses Soxhlet apparatus (DBSA) for the extraction of piperine was found to be more efficient than conventional Soxhlet apparatus (SA) based on extraction time, which is 12 ± 1 h for DBSA and 22 ± 1 h for SA [27]. In the hydrotropic extraction of piperine, hydrotropes are adsorbed on the cell wall to destroy it and then the extractant gets penetrated the cell membrane, which later helps to disorganize the amphiphilic lipid bilayer and enable easy release of piperine. Extraction temperature is increased causing more lysis of the cell, and as a result, the permeability of the cell wall is enhanced for the hydrotrope solution to act on it [1]. It demonstrated selective and rapid extraction of piperine from black pepper and the recovered piperine was approximately 90% pure [28]. The enzymolysis facilitates the breakage of the Piper nigrum L cells. This accelerates the extraction, while the addition of the surfactant promotes enzymatic hydrolysis by affecting the process of adsorption and desorption of enzymes from the substrate. This could reduce the inefficient adsorption of the enzyme, leading to its inactivation due to which an increase in the yield of piperine from 0.14 to 4.42% through HPLC is observed in surfactant-assisted enzymatic extraction of piperine [29]. In microwave-assisted extraction (MAE), the microwave power and extraction temperature are two important factors to be considered seriously as the extraction yield increases proportionally to the power increase until the increase becomes insignificant or the yield declines. Through MAE, an 85% pure piperine with a yield of 45% in 4 h was observed [1, 30].

**Table 4:** Methods of piperine extraction from black and white pepper

Methods	Apparatus used	Extraction yield & (extraction time)	Solvent used	Advantage	Disadvantage	References
CHE	Soxhlet apparatus	3.2% 2 h	95% Ethanol,10% aq. Potassium hydroxide	Simple, low cost, no filtration is required after leaching	A long time of extraction and a large amount of extractant is required	Luque de Castro and Priego-Capote [175], Shingate et al. [25]
Reflux Extraction	RBF, condenser, Buchner funnel, etc.	5% 20 min	Dichloro-methane, Acetone and Hexane	More efficient than percolation or maceration and requires less extraction time and solvent	Cannot be used for thermolabile natural products	Shingate et al. [25], Zhang et al. [176]
Cold Maceration	Separating funnel, Rota evaporator, etc.	4.6% –	GAA, chloroform, 10% sodium bicarbonate, toluene, ethyl acetate, sodium hydroxide, diethyl ether	Yield is high, pure, and crystallizable as compared to the above two methods	Complex and time-consuming	Shingate et al. [25]
EASC-CO_2_ Extraction	SPEED SFE 2, Ice-bath	0.88–1.38 mg/g dry black pepper 2.25 h	α-Amylase, CO_2_, methanol	Efficient	Expensive	Dutta and Bhattacharjee [177, 178]
ILUA Extraction	KQ-100DA and KQ-500 ultrasonic water baths (Kunshan, Jiangsu, China), Acquity™ UPLC (Waters, Milford, MA, USA)	3.57% 30 min	1-Alkyl-3-methylimidazolium ionic liquids, deionized water, methanol	High extraction efficiency, and less extraction time	Expensive	Cao et al. [179]
SLDE	Naviglio Extractor®	317.7 mg/g 3 h	96% Ethanol	Simple application, exhaustion in a short period, production of high-quality extracts	Expensive	Gigliarelli et al. [180], Naviglio et al. [181]
SMUAE	Microwave oven (CQ4250, Samsung) Ultrasonic bath (Elmasonic S10H)	46.6 mg/g 31 min	Ethanol, Methanol, Acetone, Dichloromethane Potassium hydroxide, Hexane, Acetonitrile	Increased extraction efficiency	Not suitable for thermolabile natural products	Gorgani et al. [1, 182], Zhang et al. [176]
MAE	IFB domestic microwave oven (model Neutron)	45% 4 h	Petroleum ether, water	Simple, rapid, and reliable	Not suitable for thermolabile natural products	Raman and Gaikar [30]
UAE	–	0.58% w/w 18 min	Ethanol, hexane, and acetone	Short running time, higher extractive yield, controllable parameters	Small particle size, more filtration steps	Shityakov et al. [183]
SFE	–	90–96% w/w 2–5 h	Liquid carbon dioxide	Efficient, selective, clean, fast	High cost, less pressure-resistant	Shityakov et al. [183]

### Pharmacokinetics and brain uptake distribution of piperine

Piperine (30 mg/kg, p.o.) showed a high degree of brain exposure with a Kp, brain of 0.95 and Kp, uu, brain of 1.10 it also showed high-BBB penetration potential with no interaction with efflux transporter and suggested that efficient brain uptake of piperine is due to its very limited liver metabolism evidenced by its much lower intrinsic clearance in the liver. The maximum brain concentration of piperine (20 mg/kg, i.p.) was found to be 51 ± 9 ng/g after 3 h, which could later be increased to 121 ± 7 ng/g after formulating piperine (18 mg/kg, i.p.) into solid lipid particles [31, 32]. Half-life (t_1/2_) of piperine in humans is about 13.2–15.8 h, suggesting that it has a long elimination time in the human body [31, 32]. To extrapolate the molecular mechanism of piperine, researchers are trying to explore the pharmacokinetics profile and brain uptake of piperine as a single drug and in combination with other (Table 5) [33]. Tables 6 and 7 list pharmacokinetic parameters of piperine in the human body and rodents. It was demonstrated that piperine (20 mg/kg, p.o.), when administered in conscious rats, gets absorbed rapidly through the g.i.t and could be detected in plasma within 15 min after administration. However, its metabolites were not excreted in the biliary excretion, which will be the topic of future research. In another study, it was found that Cmax in plasma assay of piperine in Wistar rats at a dose of 10 mg/kg to be about 59 ng/mL and t1/2 to be about 6 h [34]. Piperine demonstrated an unexplored effect on the oral bioavailability and intestinal permeability of cyclosporine A by modulating the P-gp (T. [31, 32]. Piperine also induces acidity by stimulating the histamine H_2_ receptors [35]. Piperine can enhance cannabinoid absorption even in chronic consumption [36]. The plasma concentration of sodium valproate (SVP) was enhanced to 14.8‑fold when SVP was administered with piperine, and a 4.6‑fold increase in the AUC of SVP + piperine was also seen [37]. Piperine combined with oxyresveratrol led to an 1.5-fold increase in the *C*_max_ & AUC, with a shorter *T*_max_ from 2.08 to 1.30 h; it is excreted in an unchanged form through the urinary route [38].

**Table 5:** Pharmacokinetics effect of piperine on different drugs

Drug	Dose (Piperine + Drug, duration)	ROA	Methods of detection	Plasma level	References
Propranolol	20 mg + 40 mg, 7 days	oral	Spectrofluorimetric method	1000–1200 ng mL^−1^ h	Bano et al. [184]
Theophylline	20 mg + 150 mg, 7 days	Oral	EMIT	*80–90 μg mL^−1^ h	Bano et al. [184]
Diclofenac	20 mg + 100 mg, 10 days	Oral	NCAM, Phoenix WinNonlin 6.2 software	7.09–11.81 μg mL^−1^ h	Satish Kumar Bedada et al. [155, 156]
CBZ	20 mg + 200 mg, 10 days	Oral	NCAM, Phoenix®, WinNonlin 6.4® software	40–70 μg mL^−1^ h	Bedada et al. [155, 156]
Emodin	20 mg/kg + 20 mg/kg, 1 day	Oral	LC–MS/MS	1913–2555 ng mL^−1^ h	Di et al. [185]
Linarin	20 mg/kg + 50 mg/kg, 1 day	Oral	NCAM, DAS 2.1.1 Software, ANOVA	240–934 ng mL^−1^ h	Feng et al. [186]
Curcumin	In rats-20 mg/kg + 2 g/kg, 1 day In humans- 5 mg + 500 mg, 1 day	Oral	MIM, PHARMKIT computer programme with SIMPLEX algorithm	3.33–3.95 μg mL^−1^ h 0.07–0.09 μg mL^−1^ h	Shoba et al. [187]
Cannabidiol	10 mg/kg + 15 mg/kg, 10 days	Oral	NCAM, WinNonlin® (version 5.2, Pharsight, Mountain View, CA)	Acute- 576–610 Ng mL^−1^ h Chronic- 722–896 ng mL^−1^ h	Izgelov et al. [36]
Fexofenadin	10 mg/kg + 10 mg/kg 10 mg/kg + 5 mg/kg, 1 day	Oral oral + IV	NCAM, WinNonlin® (version 5.2, Pharsight, Mountain View, CA)	687–1353 ng mL^−1^ h 5670–9830 ng mL^−1^ h	Jin and Han [188]
Sodium valproate	5 mg/kg + 150 mg/kg, 1 day	Oral	NCAM, trapezoidal method	1024 μg mL^−1^.h	Parveen et al. [37]
OXR	10 mg/kg + 100 mg/kg 1 mg/kg + 10 mg/kg, 1 day	Oral IV	NCAM, PK Solution 2.0 software (Summit Research Service)	9375.27 ± 1974.32 μg h/L 1471.00 ± 1945.62 μg h/L	Junsaeng et al. [38]

^*^Serum concentration

**Table 6:** Pharmacokinetic parameters of piperine in human body

Route of administration	Dose (mg)	*C*_max_ (ng/mL)	*T*_max_ (h)	AUC_0−∞_ (μg h/mL)	*t*_1/2_ (h)	References
Oral	24	3.77 ± 1.63	2.15 ± 1.21	58.41 ± 23.50	8.74 ± 8.95	Itharat et al. [189]
Oral	20	290.00 ± 42.47 595.4 ± 108.6	– –	59.32 ± 10.82 15.79 ± 50.50	13.26 ± 1.91 15.82 ± 4.95	Wen-xing [190]
Oral	20	290 ± 40	3.50 ± 1.78	5.93 ± 1.08	13.3 ± 1.9	Ren and Zuo [191]

**Table 7:** Pharmacokinetic parameters of piperine in rodents

Route of administration	Dose (mg/kg)	*C*_max_ (μg/mL)	*T*_max_ (h)	*t*_1/2_ (h)	AUC_0-∞_ (μg h/mL)	References
Oral	20	1.10	2.00	1.27	7.20	Ren and Zuo [191]
Oral	54.4	4.29 ± 0.97	2.45 ± 2.12	4.10 ± 0.94	23.1 ± 0.1	
Intravenous	10	2.90	–	8	15.6	
Intravenous	3.5	5.90 ± 1.76	–	1.68 ± 0.40	3.80 ± 0.84	
Intra-peritoneal	20	0.051 ± 0.009	3.00 ± 0.17	-	1.22	

### Enhancement of bioavailability by nanoformulations of piperine

**Fig. 5:**
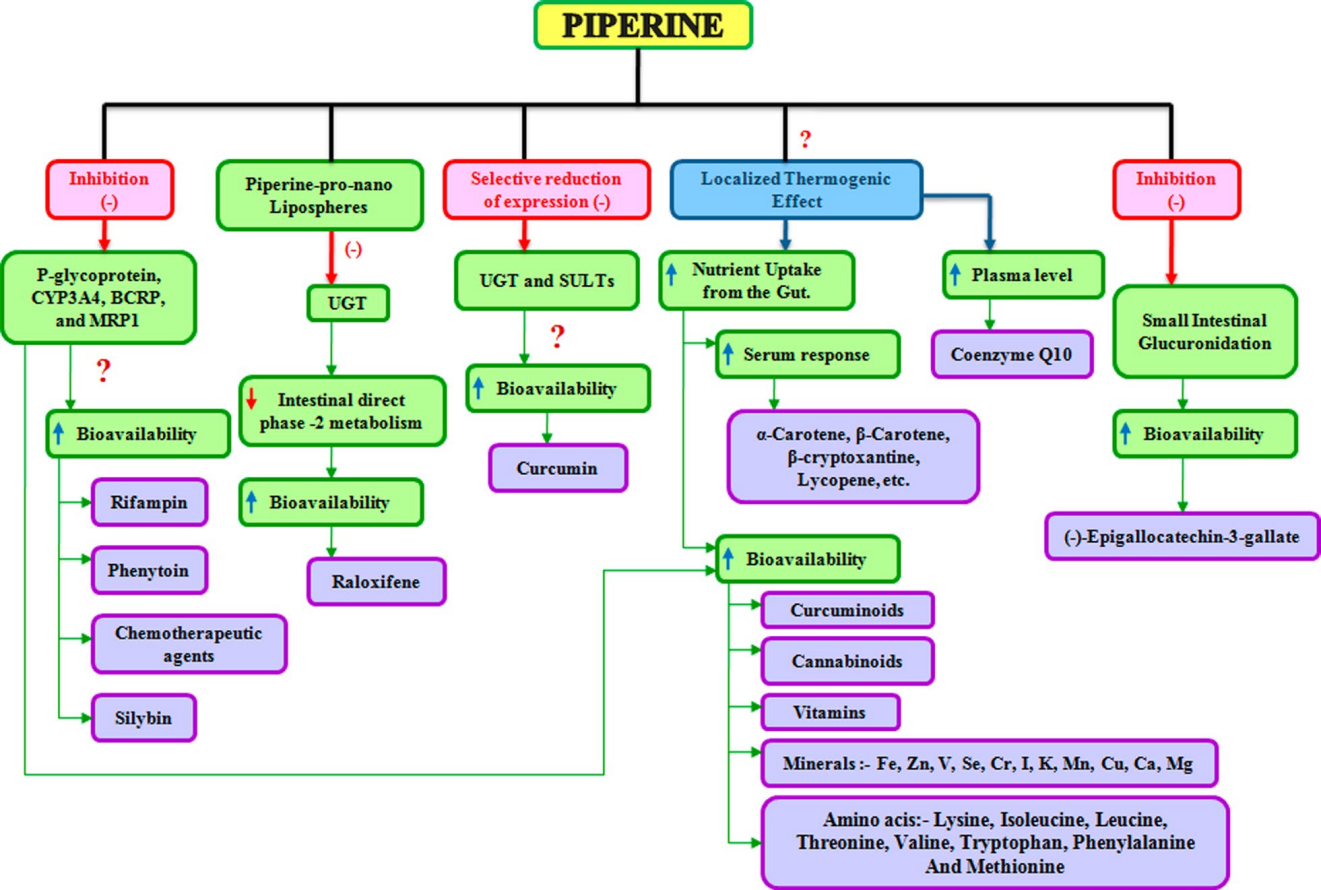
Mechanistic effect of piperine for bioavailability enhancement of various drugs with pleiotropic mechanism

Pure piperine, despite multiple biological actions, has poor water solubility and low bioavailability; thus, a modified drug-delivery system is utilized to deliver piperine in inappropriate amounts. Despite this, there are few possible explanations for the bio-enhancing property of piperine (Fig. 5). The relative bioavailability of piperine-SR-pellets is 2.70-fold higher than that of the pure piperine and a 1.62-fold compared with that of piperine solid dispersion and a 3.65-fold higher oral bioavailability as a nanosuspension than its coarse suspension [39],Y. [40, 41]. The studies provide evidence that piperine enhances the bioavailability of many compounds; the serum response of β-carotene is increased by 60% when supplemented with piperine through the oral route [42]. Piperine also increased the bioavailability of silybin by 146–181% and contributed to enhance the therapeutic effect in CCl_4_-induced acute liver-injury rat model [43]. For raloxifene in pro-nano lipospheric form with piperine, it provides a twofold increase in the oral bioavailability [44].

Piperine in combination with curcumin loaded in the emulosome is reported to achieve a sixfold increase in caspase-3 activity and is found to be most effective in the inhibition of cell proliferation [45]. A new mechanism has been introduced by which piperine enhances the bioavailability of curcumin by selectively reducing the expression of uridine diphosphate glucuronosyltransferase (UGT) and sulfotransferase (SULT) [46].

Supplementation of iron (Fe) with piperine orally benefitted the absorption of Fe and could be potentially helpful in the treatment of anemia, but an investigation is needed in this regard [47]. (−)-Epigallocatechin-3-gallate (EG) obtained from *Camellia sinensis* (green tea) is reported for its chemopreventive activity in animal models of carcinogenesis, piperine was also reported to enhance its bioavailability by 1.3-fold as compared to EG alone [48]. The combination of paclitaxel and piperine was found to improve the bioavailability of paclitaxel for triple-negative breast cancer by targeting epidermal growth factor receptor (EGFR) [49].

### Molecular targets of piperine in human

Piperine being a bioavailability enhancer increases plasma concentration of various drugs. It inhibits the drug-metabolizing enzymes by acting on P-gp substrate [50]. Although piperine has demonstrated its health benefits in human, its underlying mechanism remains unknown; information corroborated from the clinical trials often suffers the limitation of small-sized racial variations, typical considerations and geographical variations, which compromise to explore the molecular mechanism. However, cell-cycle proteins, P-gp, Rv1258c, PRPV1, Akt/mTOR signaling, AMPK-mediated activation of NLRP3 inflammasome, voltage-gated K^+^ current, IL 10, miR21, and PKCα/ERK1/2 and NF-κB/AP-1-dependent MMP-9 expression are the main targets altered by piperine (Figs. 3, 4 and 5).

The preclinical studies suggested piperine acting on various cell cycle proteins (Cyclin D, Cyclin T, CDK2 and CDK4) became a future point of intense investigation. The molecular docking analysis confirmed that piperine binds cell cycle proteins via hydrogen bonding and impaired the cell cycle progression [51]. Piperine exhibited an antitumor effect by inhibiting the S-phase by forming a hydrogen bond with Ser5 at the ATP-binding site on CDK2 protein. It interacts with the Lys8 residue in cyclin A and inhibits apoptosis by interacting with the hydrophobic groove of the Bcl-xL protein [52]. Piperine accelerates the bioavailability of phenytoin and rifampin by inhibiting the drug transporter P-gp and CYP3A4 [53]. In addition to this, ABC transporter genes are also inhibited by piperine, which pumps out several chemotherapeutic agents [54–56].

The natural product analogue Pip1 (Table 3) is found superior to piperine as for its inhibition of the P-gp function and resistance reversal in a cancer cell [50]. Rv1258c is a transporter protein that confers resistance to anti-tubercular agents like isoniazid, rifampicin, ethambutol, pyrazinamide, and p-aminosalicylic acid, approved by *in silico* studies. However, piperine in combination with these agents increases bioavailability by inhibiting the Rv1258c pump. Non-selective cation channel TRPV1 gets mildly activated by piperine, thereby inhibiting the seizure and obesity. Studies corroborated that piperine downregulated the PI3K/Akt/mTOR signaling pathway [57]. However, it reduces the MMP-9 expression in DU-145 cells. The AMPK signaling pathway plays a key role in regulating the immunological disease progression [58].

The increasing dose-dependent concentration of piperine effectively downregulates the increased NLRP3 inflammasome; however, pro-IL-18 and serum levels of IL-18 were excluded in the study [59]. Piperine induces G1 cell-cycle arrest and induces apoptosis in androgen-sensitive LNCaP and androgen-insensitive PC-3 cells by inhibiting the I_K_ [60]. Piperine in combination with curcumin and taurine decreases the plasma level of IL-10 and miR-21; however, the exact molecular mechanism of interaction needs to be investigated [61, 62].

MMP-9 is expressed abundantly in malignant tumors and contributes to cancer invasion and metastasis [63]. PKCα/ERK1/2 and NF-κB/AP-1 pathways are among the major signaling pathway that regulates tumor cell invasion. Piperine downregulated the MMP-9 expression by inhibiting PKCα/ERK1/2 and NF-κB/AP-1 pathway in PMA-induced *in vitro* tumor model [64]. It also inhibits the invasion and migration of HT-1080 cells.

PPARγ is an adipogenic transcription factor and is associated with several diseases [65]. Piperine inhibits adipocyte differentiation via an antagonistic effect on PPARɣ [66]. GM-CSF, TNF-α, MMP- 2, MMP-9, and proinflammatory cytokines like IL-1β and IL-6 are involved in cancer progression mediated by NF-κB and AP-1. Piperine inhibited the translocation of NF-κB subunits like p50, p65, and c-Rel as well as CREB, ATF-2, and c-Fos [67]. MiR-127 up-regulation is correlated with worsening of LPS-induced inflammation [31, 32]. Piperine has been showing anti-inflammatory action in the LPS-induced in vitro model of osteoarthritis by down-regulating miR-127 and MyD88 expression [68]. The wnt/β-catenin signaling is a molecular target for colorectal cancer, ovarian cancer, and HCC [69–71]. Piperine inhibits the wnt/β-catenin signaling by impairing the TCF binding to the DNA and alters the cell-cycle progression. It also decreases the metastasis in intestinal tumor cells [72]. Altered pathways are involved in many tumor developments. Piperine increases the JNK and p38 MAPK phosphorylation, thereby activating the JNK/P38 MAPK pathway and inducing apoptosis in ovarian cancer cells [73]. The anti-tumor effect of piperine is associated with stabilizing the G-quadruplex structure formed at the c-myc promoter region, which alters the transcription mechanism [74]. Piperine improves CIRI-induced injury of the ischemic penumbra region by downregulating the COX-2, NOS-2, and NF-κB [75, 76].

Piperine interacts with the _r_(CGG) ^exp^ RNA with high selectivity to the G-rich RNA motif whose expansion in 5' UTR of *FMR1* gene causes the Fragile X-associated tremor/ataxia syndrome. The transcripts of these expanded repeats _r_(CGG)^exp^ either form RNA foci or undergo the RAN translation, which in turn produces toxic proteins in the neuronal cells. Piperine is found to improve the _r_(CGG)^exp^-related splicing defects and RAN translation in the FXTAS cell model system [77].

### Different biological activities reported on piperine

#### Anticancer activity

Piperine alone and in combination with other natural or synthetic drugs has shown potential for anti-cancer activity [78]. In an *in vitro* model, piperine showed synergistic antiproliferative effects in MCF7 cell line, and it synergizes tamoxifen in combination with hesperidin and bee venom in MCF7 and T47D cell lines [79]. It lowered the LC50 value of paclitaxel (from 50 to 25 μM) and decreased the lag phase mostly during the paclitaxel action-time in an *in vitro* MDA MB-231 cell-line model. It also increased the cytotoxic and anti-proliferative effect of paclitaxel and doxorubicin when used in combination (Kanthaiah Original Research et al. [80]). In an in vivo model (EMT6/P cells were inoculated in Balb/C mice), piperine along with thymoquinone inhibited angiogenesis, induced apoptosis, and shifted the immune response toward T helper1, and further study is needed in this context [81]. *In vitro* stem cell model for breast cancer was utilized to evaluate the cancer-preventive effects of piperine and curcumin in combination therapy and the inhibition of mammosphere formation, serial passaging, aldehyde dehydrogenase (ALDH +) breast stem cells in both normal and malignant breast cells, and inhibition of Wnt signaling was observed [82]. Proliferation and induced apoptosis through caspase-3 activation and PARP (*Poly (ADP-ribose) polymerase*) cleavage were strongly inhibited by piperine, thereby inhibiting the HER2 gene expression at the transcriptional level. Pretreatment with piperine also accelerated sensitization to paclitaxel killing in HER2-overexpressing breast cancer cells [83]. Piperine causes G1 phase cell cycle arrest and apoptosis in SK-MEL 28 and B16-F0 cell lines via the activation of checkpoint kinase 1 followed by downregulation of XIAP, full-length Bid (FL-Bid), and cleavage of Caspase-3 and PARP [84]. Multidrug-resistant cancers were targeted and treated by curcumin–piperine dual drug-loaded nanoparticles [85]. Guar gum microvehicle loaded with thymoquinone and piperine exhibited low median lethal dose (LD50) value against human hepatocellular carcinoma cell lines [86]. Piperine-free extract of *Piper nigrum* exhibited anticancer effects on cholangiocarcinoma cell lines [87]. Piperine exhibited cytoprotective. The proliferation of prostate cancer cell lines was inhibited by piperine by reducing the expression of phosphorylated STAT-3 and nuclear factor-kB (NF-kB) transcription factors [88]. Piperine-loaded core–shell nanoparticles caused a substantial change in cytotoxicity compared to free drugs, with a rise in G2/M-phase and pre-GI-phase population, CDK2a inhibition, and apoptotic/necrotic rates in human brain cancer cell line (Hs683) [89]. Piperine inhibited cell-cycle progression in rectal cancer cells by causing ROS-mediated apoptosis [90].

#### Antimicrobial activity

Piperine exhibited potential inhibitory activity against Ebola and Dengue viruses by suppressing the targeted enzymes such as *Methyltransferase* of Dengue and *VP35 interferon inhibitory domain* of the Ebola virus [91]. It also showed more affinity toward viral proteins in comparison with Ribavirin. Piperine (12.5 and 25 μg/ml) showed a twofold reduction in the MIC of ciprofloxacin (0.25–0.12 μg/ml) for *Staphylococcus aureus* (ATCC 29213), the underlying mechanism for which is stated as that piperine inhibits the ciprofloxacin efflux from bacterial cells by inhibiting the P-glycoprotein [92]. Twenty-five analogues of piperine were also found to inhibit *the Staphylococcus aureus* NorA efflux pump [93]. Piperine, along with its derivatives and analogues, exhibited Leishmanicidal activity against *Leishmania amazonensis* and *Leishmania donovani* [94, 95]. Piperine (15 μg/ml) was found to inhibit the planktonic growth and shows a stage-dependent activity against biofilm growth of *Candida albicans* (ATCC10231) by affecting its membrane integrity [96]. Amide derivatives of piperine have also emerged as potential insecticides, among which the compounds 5b and 5d are the most toxic against Brazilian insect *Ascia monuste orseis* with a mortality percentage of 97.5% and 95%, respectively [97].

#### Action on metabolic diseases

The use of piperine for reversing metabolic disease usually involves a bioavailability enhancer. Greater consumption of energy leads to adiposity and fat cell enlargement producing the pathology of obesity, which is the most significant medical problem [98, 99]. Increased fat mass is associated with risk conditions such as stroke, coronary heart disease, and type 2 diabetes mellitus known as excessive fat-related metabolic disorders (EFRMD) [99, 100]. Melanocortin-4(MC-4), a hypothalamic neuropeptide, regulates obesity by controlling the feeding mechanism via binding to the MC-4 receptor [101–103]. Increased MC-4 receptor activity leads to a decrease in appetite, increased energy expenditure, and insulin sensitivity. Studies reported that piperine (40 mg/kg) can be used as an MC-4 agonist and has potential use in improving the lipid profile [104]. In addition, piperine (50 mg/kg bw) improves insulin signaling in HFD-induced hepatic steatosis by reversing the plasma adiponectin, insulin, and glucose concentration [105]. Another study suggested that supplementation of piperine (30 mg/kg) is helpful for normalizing the blood pressure, plasma parameters of oxidative stress, and inflammation [106]. However, in a randomized controlled trial to improve the bioavailability, the curcuminoids were administered with piperine (Bioperine®) in the ratio of 100:1, an efficacious adjunct therapy for patients with metabolic diseases [14].

#### Action on neurological diseases

The most common neurological disorders where piperine has shown experimental neuroprotective potential are Alzheimer’s disease (AD), Parkinson’s disease (PD), and cognitive impairment [107–109]. Various signaling molecular pathways such as oxidative stress, ER stress, inflammation, MicroRNA, mitochondrial damage, and gut microbiota have been implicated in these diseases [107–113]. Piperine with 50 mg oral dose given to human volunteers shows plasma concentration of 5 ng/mL [10]. Therefore, piperine is likely to cross the BBB [114], and the development of its potential analogue explores the application in treating neurological disorders. Piperine analogue interacts with potential CNS target like GABAA, TRPV1 and adenosine A2A receptors and MAO-B involved in neurodegenerative disease. Other studies have shown that combinational treatment of piperine with other phytochemicals like curcumin improves cognitive impairment by decreasing oxidative stress [111, 112]. Piperines play a pivotal role in neuroprotection by reducing the inflammatory cytokine, oxidative stress, and mitochondrial impairment.

Cerebral stroke is the leading cause of death and physical disability worldwide; still, only one FDA-approved drug recombinant tissue plasminogen activator (r-tPA) is working with a low therapeutic window [115]. Co-administration of r-tPA and curcumin with piperine (20 mg) can be used to increase the therapeutic window of treatment by boosting the bioavailability of curcumin by 2000% [116]. An elevated level of proinflammatory cytokine IL-1β, IL-6, and TNF-α manifests in inflammation. Piperine is able to reduce neuronal cell death in the ischemic penumbral zone by anti-inflammatory effect [76]. Piperine is a natural bioenhancer to increase the bioavailability of phytochemicals including curcumin and resveratrol [38].

Piperine neuroprotective efficacy on neurological and cognitive disorders has been examined in the rodent model of Alzheimer, Parkinson, and epilepsy diseases [108, 109, 114, 117]. Piperine (2–5–10 mg/day body weight) may also exert neuroprotective potential by examining the locomotor activity, cognitive performance, and biochemical and neurochemical manifestation of the hippocampus [108, 118]. The oral treatment of piperine (10 mg/day bwt) enhanced the cognitive learning ability in MPTP- and 6-OHDA-induced Parkinson's mouse model [109, 114]. The antioxidant property of piperine is demonstrated by its anti-apoptotic and anti-inflammatory mechanism of the 6-OHDA-induced PD model [114]**.** Piperine exerted in vitro neuroprotective effects against corticosterone-induced neurotoxicity in PC12 cells via antioxidant and mRNA expression of BDNF [119, 120]. Therefore, these results suggested that piperine crosses the BBB [121]. However, these results of preclinical studies remain to be validated for translational effect on human subjects.

#### Action on cardiovascular disease

Piperine exhibited the cardioprotective effect by regulating lipid metabolism, inflammation, and oxidative stress. Piperdardine and piperine in equal amounts lower hypotension and heart rate [122]. Intravenous administration of piperine (1.5, 2.5, and 5.0 mg/kg) decreased the increased blood pressure in rats [123]. The *Sahatsatara* (a herbal formulation) contains piperine (1.29% w/w) caused relaxation in the thoracic aorta and showed potential for vasculoprotective effect in hypertensive and nitric oxide-impaired condition in rats [124]. Piperine (20 mg/kg) exhibited significant cardioprotective ability in combination with curcumin (50 mg/kg) [125]. Piperine exhibited a vasomodulatory and blood pressure-lowering effect that could be mediated via the Ca2 + channel [126]. Piperine upregulates the ABCA1 and aids in promoting the cholesterol efflux in THP-1-derived macrophages, which later inhibits calpain activity, which indicates that piperine is a good candidate for further exploration in atherosclerosis and cardiovascular diseases [127].

#### Anti-inflammatory action

Piperine has been employed in various animal models like carrageenan-induced rat paw edema, cotton pellet granuloma, croton oil-induced granuloma pouch, formalin-induced arthritis, high fat diet-induced inflammation in subcutaneous adipose tissue, and another model like IL-1β induced expression of inflammatory mediators and ultraviolet B (UV-B)-induced inflammatory responses in the human skin for anti-inflammatory activities [128–133]. The suppression of activated phosphorylated p38, JNK, and AP-1 as well as the levels of COX- 2/PGE2 and iNOS synthesis was seen after pretreating the HaCaT keratinocyte cells with piperine prior to UV-B treatment [129]. A recent study showed that bioperine improved the bioaccessibility and in vivo anti-inflammatory activity of carrageenan-complexed piperine in Wistar rats by revealing a better bioaccessibility (*C*_max_ = 0.34 μg/ml; *T*_max_ at 30 min) of the carrageenan-complexed piperine than that of the isolated piperine (*C*_max_ = 0.12 μg/ml, *T*_max_ at 60 min) [132]. The percentage inhibition of inflammation was considerable at 56% for the carrageenan-induced paw edema model and 40% for the formalin-induced arthritis model; however, in the cotton pellet-induced granuloma model, it was only 10% [131]. Piperine in combination with curcumin at nutritional doses was able to reduce the expression of the inflammatory cytokine in the adipose tissue, indicating that it could be utilized in the treatment of inflammatory conditions in metabolic disorders related to obesity [130]. It has promising activity in the reversal of hepatotoxicity in combination with *Aegle marmelos* leaf extract; it potentiates the antioxidant and anti-inflammatory properties of *A. marmelos* [134]. It effectively abrogated the IL-1β-induced over-expression of inflammatory mediators by inhibiting the production of PGE2 and nitric oxide induced by IL-1β; in addition, it decreased the IL-1β-stimulated gene expression and production of MMP-3, MMP-13, iNOS, and COX-2 in human osteoarthritis chondrocytes; it also inhibited the IL-1β-mediated activation of NF-κB by suppressing the IκBα degradation in the cytoplasm [133]. Apart from its own anti-inflammatory activity, it is also found to enhance the anti-inflammatory activities of Thymoquinone [135]. Piperine is in combination with resveratrol decreases morbidity to some extent with little or no effect on mortality associated with lupus in Systemic Lupus Erythematosus (SLE) [136].

#### Action on reproductive organs

Piperine showed inhibitory action in the inflammation of inner lining of uterus mainly caused by *Staphylococcus aureus* [137]. Through the ERK1/2 and AKT pathways, piperine mediates the stimulation of pubertal Leydig cellular development; however, it inhibits spermatogenesis in rodents [138]. However, at a dose of 10 mg/kg, the serum gonadotropin concentration increases, whereas testosterone concentration decreases [139]. It impaired reproductive function via altered oxidative stress by increased expression of Caspase-3 and Fas protein in testicular germ cells [140]. It is reported to decrease the antioxidant activity of enzymes and sialic acid levels in the epididymis, and thus, reactive oxygen species (ROS) level increases that could potentially harm the epididymal environment and sperm function [141]. Piperine could be a lead molecule to develop reversible oral male contraceptive; however, further evidences are needed to be investigated.

#### Role of piperine on gut microbiota

Microbiota and host form complex super organism in which a symbiotic relationship confers the benefits of the host in many key aspects of life. Understanding the healthy microbiome (totality of microbes) in the human microbiome project has the major challenge and needs to decipher after the oral administration of certain phytochemicals such as piperine, lycopene, and curcumin. Piperine was tested against various culture media like *Prevotella bryantii* (B14), *Acetoanaerobium sticklandii* (SR), *Bacteroides fragilis* (ATCC 25285), *Clostridioides difficile* (ATCC 9689) among which piperine showed inhibitory action against only *B. fragilis* at concentrations ≥ 0.10 mg mL^−1^ (105 cells mL^−1^) [142]. Piperine with curcumin displayed an average of 69% increase in the species detected in gut microbiota [143]. There is an unmet need to explore the potential interaction of piperine with another nutrient by using LC–MS/MS [144]. LC–MS/MS is a technique available for simultaneous detection of degraded microbial metabolites of piperine. It was revealed by HPLC analysis that tetrahydro curcumin (235 ± 78 ng/ 100 mg tissue) was present in the adipose tissue after supplementing Curcuma-P® (extract rich in curcumin and associated with white pepper) for 4 weeks [130].

### Toxicological effect of piperine

Spices and herbs have been consumed for centuries either as food or remedial necessity. The potential health benefits of the phytochemicals from these herbs could become toxic depending on the dose of exposure and may exhibit toxic effects [145]. Piperine, when administered IV, is more toxic as compared to IG, SC, and IM. The less toxicity of piperine through the IG route is suggested as for its insolubility or chemical instability in the stomach. Thereby, piperine induces hemorrhagic ulceration in the stomach and mild-to-moderate enteritis in the SI and histopathologic lesions in the g.i.t., suggesting that piperine has a local and direct effect on the gastrointestinal lumen. The LD50 values in adult male mice for a single dose of piperine through i.v., i.p., s.c., i.g., and i.m. administration are about 15.1, 43, 200, 330, and 400 mg/kg body wt, respectively [146]. Piperine’s toxicity affects mainly the reproductive system [147]. Piperine (10 mg/kg, p.o.) induced an increase in serum gonadotropins and a decrease in intratesticular testosterone in male albino rats; reports were also there that piperine interferes with crucial reproductive events in a Swiss albino-mammalian model [148].

### Piperine as a repurposing molecule for reversing the COVID-19 pandemic

Healthy gut microbiota helps to increase the immune system of COVID-19 patients. There is unmet need to identify the different microbial metabolites present after the degradation of piperine and other plant-derived molecules by using LC–MS/MS. Microbial metabolites have an ability to cross the BBB and provide pleiotropic effects on the brain and other organs by altering the gene expression. Healthy gut microbiome identification in stool samples of COVID-19 patients may be a better approach for precision medicine by utilizing Fecal Microbiota Transplantation (FMT) technologies for COVID-19 patients. Black pepper consumption, besides its immunomodulatory functions, may also aid in combating SARS-CoV-2 directly through possible antiviral effects [149]. It has recently been reported that piperine has demonstrated binding interactions toward the spike glycoprotein and ACE2 cellular receptor for SARS-CoV-2. The interactions of hydrogen bonds with Gly399, His401, Glu402, Arg514, Arg518 were found significant by forming one predictable hydrogen bond with each amino acid residue [150]. Piperine interacts with the main protease at the docking score of -90.95 and binding energy score of -78.10 kcal mol^−1^, forming one hydrogen bond with His41; other stabilizing interactions include π -sulfur, π–σ, π–π T-shaped, and alkyl interactions. Piperine with a binding affinity of −6.4 kcal mol^−1^ forms hydrogen bond interaction with GLY164 and GLY170; its binding process is also governed by van der Waals interactions with ARG71, TYR121 (TYR453), TYR163 (TYR495), and ASN169 (ASN501) of SARS-CoV-2 receptor-binding domain spike protein (RBD Spro). The major stabilizing interactions of piperine with SARS-CoV-2 RBD Spro were by covalent hydrogen bonding, π–π T-shaped, and van der Waals force of interactions [151]. Piperine acts on the Nsp15 viral protein and inhibits SARS-CoV-2 replication [152, 153]. Furthermore, binding chemistry of piperine and curcumin via π–π intermolecular interactions enhances curcumin’s bioavailability, which facilitates curcumin to bind RBD Spro and ACE-2 receptors of host cell, thereby inhibiting the entry of virus inside the host [152, 153].

## Conclusion

Since its identification in 1820, piperine pleiotropic activities have been reported in many studies. However, most of the discussions are based on preclinical as well as in vitro model systems. As summarized in this review, piperine exhibits significant preclinical activities against a number of human diseases including cancer and inflammatory disorders. A few potential molecular targets were explored in the context of different diseases. However, some targets remain unexplored for the DAB-2 gene in the TGF-β pathway in chronic kidney disease. The underlying mechanism of its efficacy against different ailments and chronic illnesses seems to be due to its ability to modulate many different signaling pathways. Bioavailability enhancement by retarding the glucuronidation reactions, affecting certain proteins and enzymes, and increasing the nutrient uptake from the gut is among the few explanatory findings in the scope of its bioenhancer properties. Future research is needed to explore the different metabolic products produced from the gut microbiota after the microbial degradation of piperine and its related isomers. These microbe-mediated products may play a contributing factor for the toxicity of different organs.

Among all the clinical trials done on piperine, it was used either alone or in combination with other drugs, and the safe dose reported for action was 5 mg/day. A threshold of toxicity of 50 mg/kg bw/day is proposed for piperine. It is also used as a repurposed medicine to explore the inhibitory action on new molecular targets in the context of COVID-19, and only a few computational studies have been able to produce satisfactory results; however, *in vivo* models should be designed to provide thorough evidence. Further studies are needed to explore the role of other isomers isolated from black and white pepper against different targets of COVID-19 pathophysiology.

Since piperine has been consumed for centuries; the immunomodulatory action and lipid-lowering effect on metabolic diseases including cardiovascular diseases were discussed in this review. In Langendorrf's rabbit heart preparation, piperine caused partial inhibition and verapamil caused complete inhibition of ventricular contractions and coronary flow.

Piperine stimulates the digestive capacity by activating the release of digestive enzymes from the pancreas. However, the effect of piperine on the gut microbiota has been explored on a very limited scope, and therefore, it is suggested that rigorous exploration is needed in this context. The effects of piperine on kidney-related diseases need to be studied since it has a very little published establishment in this scope. The synergistic effects, as well as the combinatorial combination of piperine and other phytochemicals, should be explored for other diseases.

Piperine treatment has also been evidenced to decrease lipid peroxidation and beneficially influence the cellular thiol status, antioxidant molecule, and antioxidant enzymes. Work has been done on a computational scope for a nanoformulation incorporated in combination with piperine for human neuroblastoma SH-SY5Y cells; the conclusive results were satisfactory to have an augmented antioxidant effect on an Alzheimer’s model in vitro; however, animal-based models are needed to provide further evidence.

Regardless of all these reports, it is not yet prescribed for human use as for its limited number of clinical trials. In combination, piperine alters the metabolism and bioavailability of co-administered drugs. The number of publications on this molecule continues to increase with few clinical trials that are still ongoing. As we gather more information on the health benefits of piperine, it is more likely that the medicinal utility will be widely accepted.

## Data Availability

None.

## Abbreviations

ABSIRC: Ablon Skin Institute and Research Center
AIDS: Acquired immunodeficiency syndrome
Akt: Protein kinase B
AMPK: AMP-activated protein kinase
AP-1: Activated protein-1
AP-1: Activator protein 1
ATF-2: Activated transcription factor-2
ATP: Adenosine triphosphate
Bcl-xL: B cell lymphoma-extra large
BCRP: Breast Cancer Resistance Protein
BCRPL: Bioserve Clinical Research Pvt Ltd
BH: Baqiyatallah Hospital
BS: Bladder Spasm
BUC: Baqiyatallah University Clinic
BUMS: Baqiyatallah University of Medical Sciences
CA: California America
CBZ: Carbamazepine
CDK2: Cyclin-dependent Kinase 2
CDK4: Cyclin-dependent Kinase 4
CHE: Continuous Hot Extraction
CIRC: Chemical Injuries Research Center
CIRI: Cerebral ischemic-reperfusion induced
CKD: Chronic kidney disease
c-myc: Cellular myelocytomatosis
COX-2: Cyclooxygenase-2
CREB: CAMP-response element binding protein
CYP3A4: Cytochrome P450 3A4
DNA: Deoxyribonucleic acid
DRBCPHU: David R. Bloom center for pharmacy at The Hebrew University
EASC-CO_2_: Enzyme-assisted supercritical carbon dioxide
EMIT: Enzyme-multiplied immunoassay technique
ERK1/2: Extracellular-signal-regulated kinase ½
FMR1: Fragile X mental retardation 1
FXTAS: Fragile X-associated tremor/ataxia syndrome
GAA: Glacial acetic acid
GM-CSF: Granulocyte–macrophage colony-stimulating factor
GPLRU: Gastrointestinal Physiology Laboratory and in the Radiology Unit
HCC: Hepatocellular carcinoma
HIVS: Human Immunodeficiency Virus Seronegativity
HMO: Hadassah Medical Organization
HPLC: High-performance liquid chromatography
HSE: Hydrotropic Solubilization Extraction
IL-10: Interleukin-10
IL-12p40: Interleukin-12p40
IL-18: Interleukin-18
IL-1β: Interleukin-1β
IL-6: Interleukin-6
IL-8: Interleukin-8
ILUA: Ionic liquid-based ultrasonic-assisted
IUMS: Isfahan University of Medical Sciences
IV: Intravenous
JNK: C-Jun N-terminal kinase
KOA: Knee Osteoarthritis
LC–MS/MS: Liquid chromatography with tandem mass spectrometry
LNCaP: Lymph Node Carcinoma of the Prostate
LPS: Lipopolysaccharide
Lys8: Lysine8
MAE: Microwave-assisted extraction
MDR: Multi-drug resistance
MIM: Model-independent method
MiR-127: MicroRNA-127
miR-21: MicroRNA-21
MMP2: Matrix metalloproteinase 2
MMP-9: Matrix metalloproteinase-9
MN: Malignant neoplasm
MRP1: Multidrug resistance protein 1
MS: Multiple sclerosis
MS: Muscular spasticity
mTOR: P-mammalian target of rapamycin
MyD88: Myeloid differentiation primary response 88
NA: Not Available
NAFLD: Non-alcoholic fatty liver disease
NCAM: Non-compartmental analysis method
NF-κB: Nuclear factor kappa B
NLRP3: (NOD)-like receptor protein 3
NOD: Neurology Outpatient Department
NOS-2: Nitric oxide synthase-2
NUMS: Neyshabour University of Medical Sciences
OA: Osteoarthritis
OD: Oropharyngeal dysphagia
OXR: Oxyresveratrol.
P38 MAPK: P38 mitogen-activated protein kinase
p38MAPK: P38 mitogen-activated protein kinases
PC-3: Prostate cancer cell-3
PGIMER: Postgraduate Institute of Medical Education and Research
P-gp: P- glycoprotein
PKCα: Protein kinase Cα
PMA: Phorbol-12-myristate-13-acetate
PNS: Peripheral nervous system
PPARɣ: Peroxisome Proliferator-Activated Receptor Gamma
RAN: Repeat-associated non-ATG
RBF: Round bottom flask
ROA: Route of administration
Ser5: Serine5
SFE: Supercritical fluid extraction
SLDE: Solid–liquid dynamic extraction
SMUAE: Sequential microwave-ultrasound-assisted extraction
SRC-SBUM: Skin Research Center, Shahid Beheshti University of Medical Sciences
T2DM: Type 2 diabetes mellitus
TAP-2: Transporter associated with antigen-presenting 2
TCF: T cell factor
TCS: Tonic–clonic seizures
TNF-α: Tumor necrosis factor alpha
TRPV1: Transient Receptor Potential Vanilloid 1
UAE: Ultrasound-assisted extraction
UCPSKU: University College of Pharmaceutical Sciences Kakatiya University
UPLC: Ultra-performance liquid chromatography
UTR: Untranslated region
Wnt: Wingless-related integration site
